# Concordance analysis of dermoscopic features between five observers in a sample of 200 dermoscopic images^⋆^

**DOI:** 10.1016/j.abd.2020.12.010

**Published:** 2022-03-07

**Authors:** Enrique Rodríguez-Lomba, Belén Lozano-Masdemont, Lula María Nieto-Benito, Elisa Hernández de la Torre, Ricardo Suárez-Fernández, José Antonio Avilés-Izquierdo

**Affiliations:** a Department of Dermatology, Hospital General Universitario Gregorio Marañón, Madrid, Spain; b Department of Dermatology, Hospital Universitario de Móstoles, Madrid, Spain

Dear Editor,

Dermoscopy is a non-invasive technique that has been proven in recent metanalysis to increase the accuracy in the diagnosis of cutaneous melanoma compared to the naked eye examination. However, its application can be considered subjective and too observer-dependent, with a heavy influence by previous experience. To this date, few reproducibility studies focusing on interobserver agreement of dermoscopic images have been published. Most of them are based on the evaluation of <50 cases by each observer.^1^, ^2^, ^3^ Furthermore, since the Internet Consensus Meeting of 40 dermoscopy experts in 2003, a few novel dermoscopic features have been described that were not evaluated.^1^

The aim of this project was to analyze and determine the reproducibility of the analysis of both classic and novel dermoscopic features for the diagnosis of melanoma in 200 dermoscopic images by five blinded observers (E.H.T., L.N.B, JAAI, BLM, ERL). Previous experience on dermoscopy was >10 years (1 observer), >5 years (2 observers), and <5 years (2 observers).

A retrospective evaluation of dermoscopic images collected from the database of the Melanoma Unit in our department was performed. Images were obtained using a digital polarized dermoscopy system (Dermlite Photo II Pro HR® [3Gen®, San Juan Capistrano, CA, USA] and an Olympus E-420® camera [Olympus, Tokyo, Japan]). Lesion diameter had to fit in the picture to be included. Cases without histopathological confirmation, melanoma metastases, or melanomas of special sites (facial, acral, nail, genital, or mucosal melanoma) were excluded. The distribution of diagnosis in our sample is shown in Table 1.

**Table 1:** Distribution of diagnosis in the 200 cases for evaluation.

Diagnosis	n	%
Melanoma	99	49.5
Melanocytic nevus	45	22.5
Melanocytic nevus with histopathological atypia	22	11.0
Blue nevus	5	2.5
Spitz nevus	4	2.0
Basal cell carcinoma	14	7.0
Seborrheic keratosis	5	2.5
Dermatofibroma	2	1.0
Squamous cell carcinoma	2	1.0
Other neoplasms	2	1.0
**Total**	200	100

The following dermoscopic features were analyzed: colors (light brown, dark brown, black, blue/gray, red/pink, white), asymmetry of color/structures, atypical, pigmented network, irregular globules, streaks, irregular blotches, shiny white streaks, negative pigment o brown areas, blue-black pigmentation, milky-red areas, rainbow pattern, pseudolacunae, ulceration, and irregular vessels. Data were analyzed using SPSS version 22.0 (Chicago, IL, USA). Interobserver agreement between the five dermoscopists was evaluated using the Fleiss’ Kappa statistic test.

The results of the concordance analysis are shown in Table 2. Most of the dermoscopic features ranged Kappa values between 0.3‒0.5, which can be considered fair to moderate. Asymmetry of color and structures (yes/no) showed a moderate agreement (0.46‒0.49), slightly higher than the same evaluation considering one or two axes. The presence of more than three colors presented the moderate agreement as well (0.46). The melanoma-specific structures that showed more consistency were the shiny white streaks (up to 0.55), while structureless brown areas had the worst results (0.05). A few features that have been recently described, such as prominent skin markings and blue-black pigmentation, showed a very poor correlation (0.23 and 0.18, respectively) between all observers.

**Table 2:** Interobserver agreement of colors and dermoscopic structures (Fleiss’ Kappa statistical test).

	*K*
Three or more colors	0.46
Color asymmetry (yes/no)	0.46
Structure asymmetry (yes/no)	0.49
Color asymmetry (0‒2 axes)	0.35
Structure asymmetry (0‒2 axes)	0.36
Shiny white streaks	0.55
Polymorphous vascular pattern	0.48
Regression	0.47
Streaks/pseudopods	0.45
Atypical pigment network	0.42
Pseudolacunae	0.41
Negative pigment network	0.40
Irregular globules	0.38
Irregular vessels	0.37
Irregular blotches	0.37
Blue-white veil	0.35
Rainbow pattern	0.34
Milky red areas	0.30
Prominent skin markings	0.23
Blue-black pigmentation	0.18
Hypopigmented areas	0.16
Structureless brown areas	0.05

Previous studies have determined that features regarding the overall organization, colors, and symmetries have a higher agreement and discriminatory power than many well-known diagnostic structures such as atypical pigment networks or irregular blotches.^1^, ^2^, ^3^ Recent dermoscopic algorithms, such as CASH and TADA algorithms, are based on this idea.^4^, ^5^ This finding has been confirmed in our concordance analysis and supports this approach. On the other hand, the low values observed in novel features such as prominent skin markings and blue-black pigmentation are noteworthy. Although these features have been well-defined, and their identification may be useful to increase the suspicion of certain lesions, their interobserver agreement might seem too low to be reliable in clinical practice. This variability is expected to be more striking, especially between non-expert dermoscopists, limiting their efficacy on a diagnosis.

Dermoscopy might be a subjective diagnostic tool, and its efficacy in detecting malignant lesions dependable on previous experience. We consider that basic algorithms for non-experts should include only dermoscopic structures that are easily identified by most dermatologists. Limitations of our study are its retrospective and single-institution design.

## Financial support

None declared.

## Authors’ contributions

Enrique Rodríguez-Lomba: Approval of the final version of the manuscript; critical literature review; data collection, analysis, and interpretation; effective participation in research orientation; intellectual participation in propaedeutic and/or therapeutic; management of studied cases; manuscript critical review; preparation and writing of the manuscript; statistical analysis; study conception and planning.

Belén Lozano-Masdemont: Approval of the final version of the manuscript; critical literature review; data collection, analysis, and interpretation; effective participation in research orientation; intellectual participation in propaedeutic and/or therapeutic; management of studied cases; critical manuscript review; preparation and writing of the manuscript.

Lula María Nieto-Benito: Approval of the final version of the manuscript; data collection, analysis, and interpretation; intellectual participation in propaedeutic and/or therapeutic; management of studied cases; critical manuscript review.

Elisa Hernández de la Torre: Approval of the final version of the manuscript; data collection, analysis, and interpretation; intellectual participation in propaedeutic and/or therapeutic; management of studied cases; critical manuscript review.

Ricardo Suárez-Fernández: Approval of the final version of the manuscript; data collection, analysis, and interpretation; intellectual participation in propaedeutic and/or therapeutic; management of studied cases; critical manuscript review.

José Antonio Avilés-Izquierdo: Approval of the final version of the manuscript; critical literature review; data collection, analysis, and interpretation; effective participation in research orientation; intellectual participation in propaedeutic and/or therapeutic; management of studied cases; critical manuscript review; preparation and writing of the manuscript; statistical analysis; study conception and planning.

## Conflicts of interest

None declared.
